# Investigating the Photovoltaic Performance in ABO_3_ Structures via the Nonlinear Bond Model for an Arbitrary Incoming Light Polarization

**DOI:** 10.3390/mi14112063

**Published:** 2023-11-05

**Authors:** Hendradi Hardhienata, Indra Ramdhani, Husin Alatas, Salim Faci, Muhammad Danang Birowosuto

**Affiliations:** 1 Theoretical Physics Division, Department of Physics, IPB University, Meranti Avenue, Wing S Building, Dramaga Campus of IPB, Bogor 16680, West Java, Indonesia; indra_indra@apps.ipb.ac.id (I.R.); alatas@apps.ipb.ac.id (H.A.); 2 ESYCOM, Université Gustave Eiffel, CNRS, CNAM, 292, rue Saint-Martin, 75003 Paris, France; salim.faci@lecnam.net; 3 Łukasiewicz Research Network-PORT Polish Center for Technology Development, Stabłowicka 147, 54-066 Wrocław, Poland; muhammad.birowosuto@port.lukasiewicz.gov.pl

**Keywords:** photovoltaic bond model, second harmonic generation, arbitrary polarization

## Abstract

ABO_3_ structures commonly known as perovskite are of high importance in advanced material science due to their interesting optical properties. Applications range from tunable band gaps, high absorption coefficients, and versatile electronic properties, making them ideal for solar cells to light-emitting diodes and even photodetectors. In this work, we present, for the first time, a nonlinear phenomenological bond model analysis of second harmonic generation (SHG) in tetragonal ABO_3_ with arbitrary input light polarization. We study the material symmetry and explore the strength of the nonlinear generalized third-rank tensorial elements, which can be exploited to produce a high SHG response if the incoming light polarization is correctly selected. We found that the calculated SHG intensity profile aligns well with existing experimental data. Additionally, as the incoming light polarization varies, we observed a smooth shift in the SHG intensity peak along with changes in the number of peaks. These observations confirm the results from existing rotational anisotropy SHG experiments. In addition, we show how spatial dispersion can contribute to the total SHG intensity. Our work highlights the possibility of studying relatively complex structures, such as ABO_3_, with minimal fitting parameters due to the power of the effective bond vector structure, enabling the introduction of an effective SHG hyperpolarizability rather than a full evaluation of the irreducible SHG tensor by group theoretical analysis. Such a simplification may well lead to a better understanding of the nonlinear properties in these classes of material and, in turn, can improve our understanding of the photovoltaic performance in ABO_3_ structures.

## 1. Introduction

**Figure 1:**
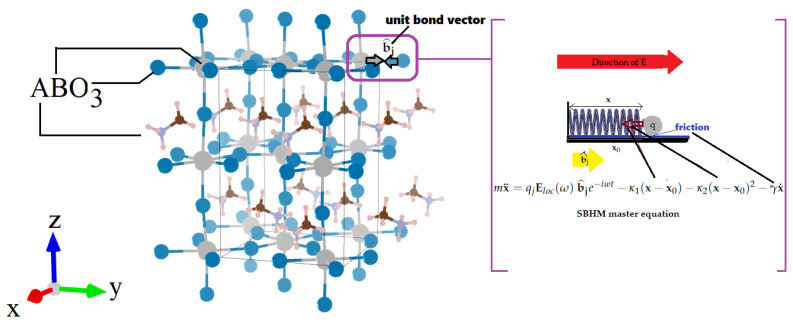
Tetragonal ABO_3_ structure under consideration and the model assumption of a damped driven anharmonic charge oscillation along the covalent bonds where the unit vector is represented by ${\hat{b}}_{j}$ (see inset). The SBHM master equation contains the local electric field $E_{loc}\left(\omega \right)$ as the driving force, the linear and SHG restoring terms, the damping constant γ, which is due to the effect of surrounding charges and is modeled as a friction force.

Perovskite ABO_3_ material is currently of high interest due to their potential role in optoelectronic and photovoltaic (PV) applications [1]. These materials possess interesting ferroelectric and PV properties [2]. One of the most potential perovskite materials with many interesting optical applications is metal halide perovskite, which can be studied using second harmonic generation (SHG) [3,4]. The tetragonal ABO_3_ structure is depicted in Figure 1. Often called perovskite, it is a specific crystal framework found in many materials. In this structure, “A” typically represents a large cation (e.g., lanthanum or barium), “B” is a smaller cation like a transition metal (e.g., titanium or manganese), and O_3_ denotes three oxygen anions forming a BO_6_ octahedron. These octahedrons are interconnected, creating a 3D network whose arrangement depends on the cation sizes and charges. Recently, ABO_3_ structures in the form of halide perovskites have acted well in outstanding solar cells, which achieved a best efficiency of 25.7% [5]. This success is partly due to the intensive study of solar cell material interface at the nanoscale, which is crucial to the photovoltaic performance. For example, it is known that a robust perovskite–substrate interface is important for realizing novel inverted perovskite solar cells (PSCs) due to the ability of charge carrier selectivity via electrostatics, energy level alignment, as well as low interfacial recombination [6]. Moreover, optimization of the perovskite interface and transport layers can enhance the photovoltaic performance of inverted perovskite solar cells [7].

Another purpose of this work is to apply the nonlinear bond model to investigate the existence of the Rashba effect in the bulk of ABO_3_ perovskite structures, which are known to reduce the expected charge-carrier mobility [8] and, therefore, can alter the photovoltaic cell performance significantly. Here, we focus our study on the tetragonal ABO_3_ perovskite structure due to the availability of rotational anisotropy second harmonic generation (RASHG) experimental data in Ref. [4], which can be tested by the bond model. It is well known that ABO_3_ metal halide perovskites, such as methylammonium lead iodide (MAPbI_3_) and formamidinium lead iodide (FAPbI_3_), can exist in three different phases depending on the temperature. Above 330 K and 300 K, MAPbI_3_ and FAPbI_3_ form a cubic structure, respectively. The tetragonal MAPbI_3_ phase —which is studied here—exists for a temperature range between 160 and 330 K, whereas for the FAPbI_3_ perovskite, the tetragonal phase exists between 130 and 300 K. Below 160 K and 130 K, MAPbI_3_ and FAPbI_3_ tetragonal phases change into orthorhombic phases, respectively [9]. The occurrence of Rashba spin–orbit coupling in such material has been found to reduce the carrier recombination rate due to the spin-forbidden transition, which affects the photovoltaic performance [10]. It has been known that the Rashba effect is due to the heavy atoms and inversion symmetry breaking inside the halide perovskite crystal [11]. However, the occurrence of the Rashba effect within hybrid organic–inorganic lead–halide perovskites (HOIP), such as MAPbI_3_, is both intriguing and a subject of ongoing debate because of uncertainties regarding the existence of symmetry breaking inside the material bulk [12].

This work can shed light on resolving this debate by performing a theoretical investigation of the nonlinear SHG dipole radiation at the nanoscale within the bulk anfd interface of tetragonal ABO_3_ perovskite and compare the results with the existing RASHG experimental data. The theoretical results can enhance the PV performance in several ways: (1) Offer a simple way to investigate the molecular structure of ABO_3_ at the surface/interface, including surface reconstruction/modification [13] and donor impurity detection, which can lead to better PV fabrication, see, e.g., Ref. [14] for the case of tunable donor detection in polymer solar cells or Ref. [15] concerning the detection of buried interfacial defects in inverted perovskite solar cells. Moreover, the model can also be applied for nanoscale and real-time molecular surface deposition monitoring [16], which is crucial in altering the PV performance. (2) Reveal whether there exists any bulk symmetry breaking, e.g., due to the cation dynamics and doping insertion, which can lead to the rise of the Rashba effect and alter the PV performance. When supported by experimental data, this can lead to a better understanding of the physical mechanism behind their PV performance.

## 2. Materials and Methods

Here, we will apply the novel nonlinear theoretical method termed “simplified-bond hyperpolarizability model (SBHM)”. SBHM is a classical phenomenological theory based on a classical Lorentz nonlinear oscillator model proposed in 2002 by Powell and Coworkers [17]. Inspired by Ewald–Oseen’s method [18,19] to calculate far-field intensities in linear optics via superposition of dipoles, they developed this principle to nonlinear optics and obtained a simpler analysis than the linear case [20]. It is an alternative approach to describe nonlinearity, in particular, second harmonic generation (SHG) from materials and interfaces. Even though it is a phenomenological theory requiring the hyperpolarizabilities to be obtained from experimental fitting, it does provide a physical framework in which nonlinear optical spectra can be represented and analyzed at the bond level in simple terms. Here, we provide a brief description of the SBHM construction to study the third-rank tetragonal ABO_3_ SHG tensor and how to produce the SHG intensity in such structures. The electron charges when driven by an incoming external field will produce linear oscillations. If the intensity of the field is large, as in the case of a femtosecond laser, the charges will also oscillate an-harmonically. Based on the electronic density orbital distribution that can be calculated using density functional theory, the charges are radiating dipoles mainly along the covalent bonds. The electrons also experience damping from their neighboring electrons; hence, the classical dynamical equations should take the form of a dimensional an-harmonically damped-driven oscillation model:

(1) $$\begin{array}{c} m\ddot{x}=q_{j}E_{loc}\left(\omega \right)\;{\hat{b}}_{j}e^{-iwt}-\kappa_{1}\left(x-x_{0}\right)-\kappa_{2}{\left(x-x_{0}\right)}^{2}-\gamma \dot{x} \end{array}$$

here x is displacement of the electron charge along the ${\hat{b}}_{j}$ bond unit vector direction, $x_{0}$ represents the charge equilibrium position, $E_{loc}\left(\omega \right)$ refers to the local field. The restoring constants $\kappa_{1}$ and $\kappa_{2}$ are the harmonic and an-harmonic spring constant. The damping constant γ is introduced in the equation to model the electron damping.

**Figure 2:**
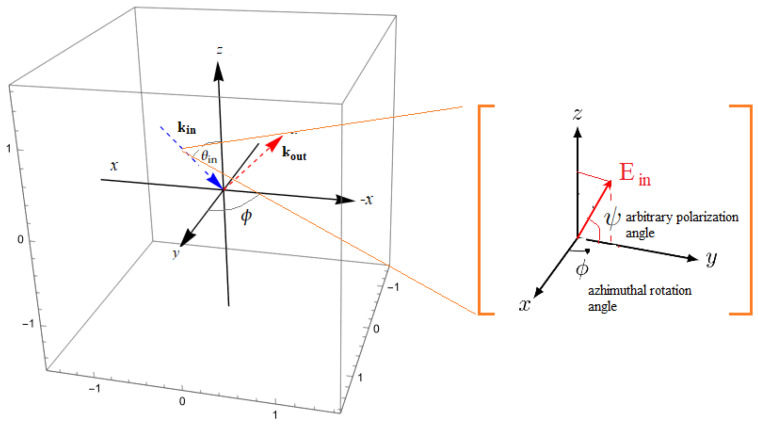
Definition of the applied coordinate system for the incoming fundamental and outgoing SHG light, azimuth (ϕ), and arbitrary polarization angle (ψ).

The applied coordinate system in our model is presented in Figure 2. The incoming fundamental and outgoing SHG fields are propagating along the xz plane. In this work, we do not limit ourselves to the four standard incoming *p* and *s*-polarized incoming fields but instead generalized the model for arbitrary input polarization:

(2) $$E_{loc}=\left(\begin{array}{c} \cos\theta_{i}\sin\psi \\ \cos\psi \\ \sin\theta_{i}\sin\psi \end{array}\right)$$

where $\theta_{i}$ is the incoming light angle relative to the *z*-plane, and ψ is the arbitrary polarization angle, which we will analyze in this work by a regular interval of 10°. From the yellow inset in Figure 2, it is obvious that when ψ= 0°, we have a fully *s*-polarized incoming field, and when ψ= 90°, we have a fully *p*-polarized incoming field.

Solving Equation (1) for $\Delta x_{1}$ and $\Delta x_{2}$, by using the assumption that *x* can be written as $x=x_{0}+\Delta x_{1}e^{-i\omega t}+\Delta x_{2}e^{-i2\omega t}+\ldots$ gives for the lowest order of approximation:

(3) $$\begin{array}{c} \Delta x_{1}=\frac{q_{j}{\hat{b}}_{j}\cdot E_{loc}\left(\omega \right)}{\kappa_{1}-m\omega^{2}-ib\omega } \end{array}$$

(4) $$\begin{array}{c} \Delta x_{2}=\frac{q_{j}\kappa_{2}\Delta x_{1}^{2}}{\kappa_{1}-4m\omega^{2}-2ib\omega } \end{array}$$

Defining the linear $p_{1j}$ and quadratic $p_{2j}$, dipole polarizations are produced by each bond $b_{j}$ as

(5) $$\begin{array}{c} p_{1j}=q_{j}\Delta x_{1}{\hat{b}}_{j}=\alpha_{1j}{\hat{b}}_{j}\left({\hat{b}}_{j}\cdot E_{loc}\left(\omega \right)\right) \end{array}$$

(6) $$\begin{array}{c} p_{2j}=q_{j}\Delta x_{2}{\hat{b}}_{j}=\alpha_{2j}{\hat{b}}_{j}{\left({\hat{b}}_{j}\cdot E_{loc}\left(\omega \right)\right)}^{2} \end{array}$$

here, $\alpha_{1j}$ and $\alpha_{2j}$ are, respectively, the microscopic linear polarizability and first-order nonlinear hyperpolarizability of the jth bond.

Therefore, the total polarization can be written as:

(7) $$\begin{array}{cc} P & =\frac{N}{V}\sum_{j}^{}p_{j}\\ \; & =\frac{N}{V}\sum_{j}^{}\left(\alpha_{1j}{\hat{b}}_{j}⊗{\hat{b}}_{j}\right)\cdot E_{loc}\left(\omega \right)\\ \; & +\frac{N}{V}\sum_{j}^{}\left(\alpha_{2j}{\hat{b}}_{j}⊗{\hat{b}}_{j}⊗{\hat{b}}_{j}\right)..\left(E_{loc}\left(\omega \right)⊗E_{loc}\left(\omega \right)\right) \end{array}$$

where *V* is the volume, *N* is the number of atomic units.

The total macroscopic linear and SHG polarization can be calculated by summing over all dipoles reminiscent of the Ewald–Oseen approach [21]. The formula of the total linear and SHG polarization is given by:

(8) $$\begin{array}{c} P={\overset{↔}{\chi }}^{\left(1\right)}\cdot E_{loc}\left(\omega \right)+{\overset{↔}{\chi }}^{\left(2\right)}..E_{loc}\left(\omega \right)⊗E_{loc}\left(\omega \right) \end{array}$$

here ${\overset{↔}{\chi }}^{\left(1\right)}$ and ${\overset{↔}{\chi }}^{\left(2\right)}$ are the first and second-order susceptibility tensors of the system. We will focus on $\chi^{\left(2\right)}$, which is related to SHG. More explicitly, from Equation (7), the SHG third-rank tensor can be obtained by the direct product of the bond unit vectors:

(9) $$\begin{array}{c} {\overset{↔}{\chi }}^{\left(2\right)}=\frac{N}{V}\sum_{j}^{}\alpha_{2j}\left(R_{z}\left(\phi \right){\hat{b}}_{j}\right)⊗\left(R_{z}\left(\phi \right){\hat{b}}_{j}\right)⊗\left(R_{z}\left(\phi \right){\hat{b}}_{j}\right) \end{array}$$

where *N* and *V* denote the total SHG dipoles, and *V* is the volume of the considered computational unit cell. In addition, to model the RASHG experiments later on, the sample is rotated along the *z*-axis to produce a polar nonlinear intensity plot. In our calculation, the azimuth angle ϕ is measured from the x-axis and attached to each bond direction by applying the rotation matrix $R_{z}\left(\phi \right)$:

(10) $$\begin{array}{c} R_{z}\left(\phi \right)=\left(\begin{array}{ccc} \cos\phi & -\sin\phi & 0\\ \sin\phi & \cos\phi & 0\\ 0 & 0 & 1 \end{array}\right) \end{array}$$

Incorporating Equation (10) into Equation (9) gives a more general formula for the nonlinear SH susceptibility in terms of the bond unit vector direct product:

(11) $$\begin{array}{c} {\overset{↔}{\chi }}^{\left(2\right)}=\frac{N}{V}\sum_{j}^{}\alpha_{2j}\left(R_{z}\left(\phi \right){\hat{b}}_{j}\left(\phi \right)\right)⊗\left(R_{z}\left(\phi \right){\hat{b}}_{j}\left(\phi \right)\right)⊗\left(R_{z}\left(\phi \right){\hat{b}}_{j}\left(\phi \right)\right) \end{array}$$

The SHG far field can be obtained via [17]:

(12) $$\begin{array}{cc} E_{ff} & =k^{2}\frac{e^{ikr}}{r}\left(\sum_{j}^{}p_{j}-\hat{k}\left[\hat{k}.\sum_{j}^{}p_{j}\right]\right)\\ \; & =k^{2}\frac{e^{ikr}}{r}\left(\overset{↔}{I}-\hat{k}\hat{k}\right).\sum_{j}p_{j} \end{array}$$

where $\overset{↔}{I}$ denotes the unit tensor, $k=k\hat{k}$ represents the outgoing light wave vector directed toward the SHG detector. It is clear that Equation (12) describes the far field produced by the sum of all the SHG dipole radiation. Due to incoming light wavelength λ being much larger than the atomic distance, the possible phase difference due to translational distance between different bond radiations can be ignored. To obtain the nonlinear SHG intensity Equation (12) is multiplied by their complex conjugate:

(13) $$I_{ff}=E_{ff}\cdot E_{ff}^{*}$$

## 3. Results and Discussion

In this section, we will present the results and discuss them accordingly. The first step in the SBHM is to develop the bond unit vector for the ABO_3_ structure and study its symmetry properties. Understanding the symmetry of a particular PV structure is very useful in interpreting the results or in simplifying the calculations. We thus begin by analyzing the surface bonds, which only consist of one atomic layer, namely, the layer on the foremost top of the ABO_3_ 3D structure in Figure 1. The bonds that are analyzed are those that form a covalent bond since it defines a “directional” orientation of the charge oscillation, e.g., along the bond. Therefore, we do not include the A large cation SHG contribution, which has been proven to be a valid assumption based on Ref. [4] and focus instead on the B cation and O_3_ anions. Due to the nature of the shared electrons in the covalent bonds, the bond unit vector arrows from the A cation and the O_3_ anions will point toward each other but are not of the same strength due to the different SHG hyperpolarizability $\alpha_{B}$ and $\alpha_{O_{3}}$, as pictured in the purple inset of Figure 1. This means that the two opposing bond vectors can be represented instead by just one bond vector represented by the “effective” hyperpolarizability:

(14) $$\alpha_{eff}=\left|\alpha_{B}-\alpha_{O_{3}}\right|$$

The utilization of an effective hyperpolarizability in SBHM has been validated for the case of zincblende structure [13]. In addition, we would like to mention that the true value of the nonlinear hyperpolarizability requires either a full knowledge of all the possible SHG sources whether it is of dipolar, quadrupolar, or spatial dispersion contribution both at the surface and bulk, which is complicated and difficult or an ab initio quantum mechanical calculation, e.g., density functional theory (DFT) [22] or polarizable quantum mechanical model [23], which is currently beyond the scope of this work. However, due to the classical nature of the phenomenological nonlinear bond model, one can have physical insight about the experimental interpretation at the nanoscale/molecular level, such as investigating the origin of the nonlinear sources and interpreting RASHG experimental data to the individual covalent bonds.

### 3.1. SHG Symmetry Analysis and Intensity

**Figure 3:**
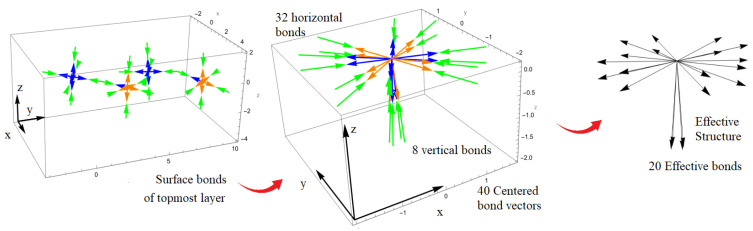
SBHM construction of the topmost ABO_3_ surface structure and their 40 centered bond vectors, which are further reduced to 20 known as the “effective” bond vectors. These 20 bonds contain the effective SHG hyperpolarizabilities $\alpha_{eff}$ and will be applied to construct the third rank tensor and the SHG intensities. Different bond vector colors are presented for distinguishing the opposite bond directions.

By looking at the surface structure symmetry in Figure 3, one can infer that 40 surface bond vectors are required to describe the surface ABO_3_ structure. These consist of the 32 bond vectors aligned slightly horizontally about the xy-axis and the 8 bond vectors aligned slightly vertically along the *z*-axis, as given by Figure 3. Indeed, when analyzed in their effective form, the number of bonds is immediately reduced to 20. The detailed orientation of each effective bond vector is given in Appendix A. Meanwhile, looking at the effective structure, one can see that it resembles a C_2*v*_ point group in group theory but now with only one fitting parameter ($\alpha_{eff}$). When the 20 bond vectors are inserted into the SBHM SHG nonlinear susceptibility in Equation (11), which already includes the rotation matrix, we obtain the “generalized” third-rank SHG tensor:

(15) $$\chi_{ijkl}=\left(\begin{array}{ccc} \left(\begin{array}{ccc} 0 & 0 & d_{1113}\\ 0 & 0 & d_{1123}\\ d_{1131} & d_{1132} & 0 \end{array}\right) & \left(\begin{array}{ccc} 0 & 0 & d_{1213}\\ 0 & 0 & d_{1223}\\ d_{1231} & d_{1232} & 0 \end{array}\right) & \left(\begin{array}{ccc} 0 & 0 & d_{1313}\\ 0 & 0 & d_{1323}\\ d_{1331} & d_{1332} & 0 \end{array}\right)\\ \left(\begin{array}{ccc} 0 & 0 & d_{2113}\\ 0 & 0 & d_{2123}\\ d_{2131} & d_{2132} & 0 \end{array}\right) & \left(\begin{array}{ccc} 0 & 0 & d_{2213}\\ 0 & 0 & d_{2223}\\ d_{2231} & d_{2232} & 0 \end{array}\right) & \left(\begin{array}{ccc} 0 & 0 & d_{2313}\\ 0 & 0 & d_{2323}\\ d_{2331} & d_{2332} & 0 \end{array}\right)\\ \left(\begin{array}{ccc} 0 & 0 & d_{3113}\\ 0 & 0 & d_{3123}\\ d_{3131} & d_{3132} & 0 \end{array}\right) & \left(\begin{array}{ccc} 0 & 0 & d_{3213}\\ 0 & 0 & d_{3223}\\ d_{3231} & d_{3232} & 0 \end{array}\right) & \left(\begin{array}{ccc} 0 & 0 & d_{3313}\\ 0 & 0 & d_{3323}\\ d_{3331} & d_{3332} & d_{3333} \end{array}\right) \end{array}\right)$$

where the nonzero tensor elements are given in Table A1 in Appendix A.

Evaluation of ϕ=0 immediately simplifies the tensor and, when compared, indeed has the same nonzero elements as a C_2*v*_ point group symmetry, exactly matching the group theory calculations in the tensor table given in Ref. [24] but now with less independent elements. As can be seen in Table A2 in Appendix A, one can obtain the non-zero tensor components as a function of the effective hyperpolarizability $\alpha_{eff}$. As is evident from the table, each coefficient’s magnitude describes the power of the nonlinear response due to an incoming fundamental field. Therefore, choosing the correct electric field polarization and material orientation ϕ can increase the SHG contribution significantly.

Furthermore, evaluation of the bulk bond vector to obtain the third rank SHG tensor analogous to the previous analysis in obtaining the surface tensor elements yields the result that all the bulk elements are zero. This can be well understood from the viewpoint of inversion symmetry. When evaluated, the reduced bulk structure has a point group symmetry of D_4*h*_, which is similar to silicon, and is centrosymmetric. In addition, based on Ref. [17], the third rank tensor element’s expression for this particular point group is zero, which confirms our findings and, thus, the tetragonal ABO_3_ bulk dipole SHG contribution is zero. However, there can be other SHG contributions in the form of bulk quadrupoles and spatial dispersion [25] within the centrosymmetric bulk. Further work is required to investigate these SHG sources using the SBHM.

**Figure 4:**
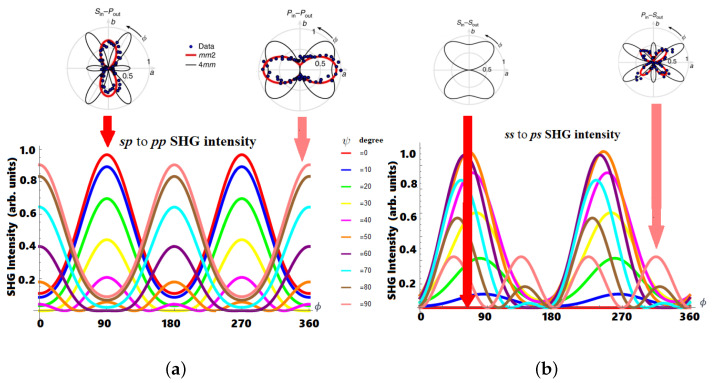
(**a**) SHG sp to pp polar plot intensities predicted by the SBHM and RASHG experimental result. (**b**) SHG ss to ps polar plot intensities predicted by the SBHM and RASHG experimental result. The experimental data were reprinted from Ref. [4].

Having analyzed the third rank SHG tensor, we can obtain the SHG intensity profile for arbitrary input polarization. We choose for the calculation an incoming fundamental-outgoing SHG light angle of $\theta_{\mathrm{in}}=\theta_{\mathrm{out}}=45^{\circ }$ on the (xz)-plane. Since the SHG signal comes from the surface, we do not account for the Fresnel coefficients, as is the case of SHG bulk dipolar contribution in zincblende structures [13]. For convenience, we set the effective SHG hyperpolarizability $\alpha_{eff}=1$ and plot the SHG intensity using Equation (7) for arbitrary light input polarization ψ variation of 10°. To better present the phase shift and compare the SHG intensity for the various incoming light polarization angles, we present the result in the form of Figure 4, where the experimental RASHG data is included to validate the SBHM model and observe whether there is a RASHBA effect from within the perovskite bulk. The result in Figure 4a shows that when the incoming light is varied from *s* to *p* for the case of a *p*-SHG outgoing wave we obtain a two-fold SHG peak with a phase difference of 90°. This peak shift is due to the inherited symmetry of the ABO_3_ surface. When an *s*-polarized field is incoming and rotated 360°, the eight vertical bonds can be seen as a symmetric inverted “V” shape thus producing a fully 2-fold SHG peak feature, whereas the slightly horizontally aligned 32 bonds will cancel out due to their “symmetry” with the direction of the electric field along the *y*-axis. However, when the incoming field polarization is shifted toward the *p*-polarization (sp to pp), the phase of the peaks is shifted 90° but still retains the 2-fold pattern. Similarly, for the ss to ps SHG polarization in Figure 4b, we can see the peak evolution from a zero-fold (ss) to a smaller fourfold peak (ps) when the incoming light is varied from *s* to *p*-polarization and the outgoing SHG field is shifted to the *s*-polarization. All the results are in good agreement with the RASHG experimental data in Ref. [4] for an mm2 space group symmetry or C_2*v*_ point group symmetry related to surface/interface SHG dipole contribution and no bulk SHG contribution (black lines), confirming the inversion symmetry of the ABO_3_ tetragonal structure. Performing a RASHG for arbitrary polarization can thus present us with more information on the true SHG sources and this work stimulates further experimental work in this area. In addition, our findings will pave the way for a better understanding and modelling of the dynamics of SHG in ABO_3_ structures. This will be especially valuable in establishing a straightforward connection between the inherent symmetry of the material and the polarization of the driving electric field. It will also enable us to investigate more complex structures, such as surface reconstruction, material impurities, and surface kinetics, using NLO techniques in the future.

### 3.2. SBHM Role in Investigating PV Efficiency: Rashba Effect

**Figure 5:**
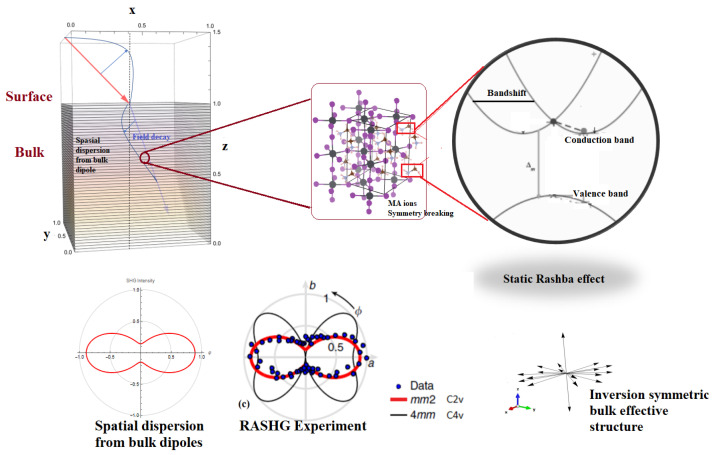
From top left to bottom left: Spatial dispersion SHG contribution in the form of bulk dipoles that radiates SHG due to the imbalance in the electric driving field. The purple inset in the top middle shows that MA ion breaking might still contribute to the SHG intensity profile, which is better resolved using an arbitrary polarized SBHM model, which includes the MA ion contribution. The bottom right picture shows that the bulk dipole bond vector structure produces a zero third-rank tensor if spatial dispersion is not included. The RASHG experiment of the pp-SHG polarization in Ref. [4] can be reproduced by SBHM using spatial dispersion.

It is clear from the obtained results that the nonlinear symmetry properties and intensity profile of ABO_3_ can be studied in a more direct and simplified way via the SBHM. Since SHG is surface sensitive and can detect nanostructural changes, this model can be used to study possible nanoscale impurities and surface reconstruction, which can alter the PV ability. Very recently, SHG has been applied to study whether a spin–orbit coupling mechanism, known as the Rashba effect, inside the bulk of MAPbI_3_ metal halide perovskite (MHP) exists [4]. The Rashba effect in such material can occur when there is symmetry breaking inside the perovskite bulk, e.g., due to the dynamics of the A site or methylammonium (MA) in the case of MAPbI_3_, which we neglected in our model, as can be seen in Figure 5. Rotational anisotropy (RA) SHG measurement in Ref. [4] shows that the SHG intensity profile matches the SBHM model of surface dipole radiation in Figure 4 and Figure 5 rather than if bulk asymmetry is assumed (black lobes in Figure 4 and Figure 5 RASHG pp-SHG intensity). Thus, our findings at the moment support the idea that there is no static bulk Rashba effect, which can alter the PV performance due to the shifts in the direct–indirect bandgap of MHP.

However, we have to state that the SHG contribution from the bulk quadrupole and spatial dispersion has not been included in our current discussion. It has been found that these two additional SHG sources can contribute to the total SHG intensity in silicon [25]. Spatial dispersion can occur because of the decay of the incoming electric field via absorption. Another effect is the SHG energy loss from electron heating due to the electric field, which has not been incorporated in the model since it does not affect the SHG intensity profile although it can decrease the SHG intensity as a whole and this is one of the reasons why we apply the arbitrary SHG intensity unit. In this work, we apply the SBHM spatial dispersion algorithm to the case of ABO_3_ and see whether their SHG intensity profile is similar to the surface dipole contribution. The spatial dispersion is straightforwardly calculated by taking the refracted angle close to the normal angle (e.g., smaller than 10°). When we set the angle close to zero, we obtain the 2-fold SHG peak lobe, as given in the bottom left picture in Figure 5 similar to the pp-polarization SHG intensity profile in Figure 4. Therefore, we can conclude that bulk dipoles can still contribute to the total SHG intensity besides the bulk quadrupole contribution contrary to the claim by Ref. [4] that there can be no bulk SHG contribution. In addition, further work on incorporating the MA ion dynamics and quadrupole contribution into the SBHM model is required to rule out whether there is truly no static bulk Rashba effect, which can affect the PV cell performance. If the modified and more complete SBHM result shows a similar RASHG profile, then there is the possibility that the static Rashba effect can still occur from the bulk of MAPbI_3_ thus invalidating the results in Ref. [4]. However, if the quadrupole and MA ion symmetry breaking dispersion profile is different than the RASHG experiment, then it supports their previous claim.

## 4. Conclusions

We have developed previous SHG SBHM models to include arbitrary input polarization for the first time to tetragonal ABO_3_ structures. We demonstrate that the SHG dipole contribution originates from the surface and is defined by a set of 20 effective bond vectors. The “generalized” third-rank SHG tensor introduced encompasses the azimuth rotation degree of freedom, revealing that the only requirement for fitting is the effective SHG hyperpolarizability $\alpha_{eff}$. Observations show a two-fold SHG peak intensity with a 90° phase shift when varying the incoming light polarization and SHG outgoing light polarization from sp to pp. Additionally, the peak transitions from a zero-fold to four-fold with a minor phase shift when the incoming light polarization changes from ss to ps. We further propose that the RASHG pp SHG polarization profile can be better modeled when spatial dispersion, or specifically contributions from bulk dipoles undergoing varying field decay along the *z*-axis, is included. Crucial future work entails the inclusion of bulk quadrupole and MA symmetry breaking, vital for determining the potential existence of a static bulk Rashba effect in ABO_3_ material bulk. Through our work, an enhanced comprehension regarding the dynamics of the SHG peak and phase shift provides deeper insights into SHG in ABO_3_ structures, with the prospects of investigating surface reconstruction, material impurities, and surface kinetics, all pivotal for optimizing PV efficiency.

## Data Availability

The data concerning all the results in this work are not publicly available at this moment but may be obtained from the authors upon request.

## Appendix A

**Table A1:** ABO_3_ surface effective bond vectors with $\beta =\frac{\pi }{2}$.

Bond Vectors	*x* Component 1	*y* Component	*z* Component
b[1]	$\frac{1}{2}\sin\left(\frac{19}{18}\beta \right)$	$\frac{1}{2}\sqrt{3}\sin\left(\frac{19}{18}\beta \right)$	$\cos\left(\frac{19}{18}\beta \right)$
b[2]	$\frac{1}{2}\sin\left(\frac{17}{18}\beta \right)$	$\frac{1}{2}\sqrt{3}\sin\left(\frac{17}{18}\beta \right)$	$\cos\left(\frac{17}{18}\beta \right)$
b[3]	$-\frac{1}{2}\sin\left(\frac{19}{18}\beta \right)$	$-\frac{1}{2}\sqrt{3}\sin\left(\frac{19}{18}\beta \right)$	$\cos\left(\frac{19}{18}\beta \right)$
b[4]	$-\frac{1}{2}\sin\left(\frac{17}{18}\beta \right)$	$-\frac{1}{2}\sqrt{3}\sin\left(\frac{17}{18}\beta \right)$	$\cos\left(\frac{17}{18}\beta \right)$
b[5]	$\frac{1}{2}\sqrt{3}\sin\left(\frac{17}{18}\beta \right)$	$\frac{1}{2}\sin\left(\frac{17}{18}\beta \right)$	$\cos\left(\frac{17}{18}\beta \right)$
b[6]	$\frac{1}{2}\sqrt{3}\sin\left(\frac{19}{18}\beta \right)$	$\frac{1}{2}\sin\left(\frac{19}{18}\beta \right)$	$\cos\left(\frac{19}{18}\beta \right)$
b[7]	$-\frac{1}{2}\sin\left(\frac{17}{18}\beta \right)$	$\frac{1}{2}\sqrt{3}\sin\left(\frac{17}{18}\beta \right)$	$\cos\left(\frac{17}{18}\beta \right)$
b[8]	$-\frac{1}{2}\sin\left(\frac{19}{18}\beta \right)$	$\frac{1}{2}\sqrt{3}\sin\left(\frac{19}{18}\beta \right)$	$\cos\left(\frac{19}{18}\beta \right)$
b[9]	$-\frac{1}{2}\sqrt{3}\sin\left(\frac{19}{18}\beta \right)$	$-\frac{1}{2}\sin\left(\frac{19}{18}\beta \right)$	$\cos\left(\frac{19}{18}\beta \right)$
b[10]	$-\frac{1}{2}\sqrt{3}\sin\left(\frac{17}{18}\beta \right)$	$-\frac{1}{2}\sin\left(\frac{17}{18}\beta \right)$	$\cos\left(\frac{17}{18}\beta \right)$
b[11]	$\frac{1}{2}\sin\left(\frac{17}{18}\beta \right)$	$-\frac{1}{2}\sqrt{3}\sin\left(\frac{17}{18}\beta \right)$	$\cos\left(\frac{17}{18}\beta \right)$
b[12]	$\frac{1}{2}\sin\left(\frac{19}{18}\beta \right)$	$-\frac{1}{2}\sqrt{3}\sin\left(\frac{19}{18}\beta \right)$	$\cos\left(\frac{19}{18}\beta \right)$
b[13]	$-\frac{1}{2}\sqrt{3}\sin\left(\frac{17}{18}\beta \right)$	$\frac{1}{2}\sin\left(\frac{17}{18}\beta \right)$	$\cos\left(\frac{17}{18}\beta \right)$
b[14]	$-\frac{1}{2}\sqrt{3}\sin\left(\frac{19}{18}\beta \right)$	$\frac{1}{2}\sin\left(\frac{19}{18}\beta \right)$	$\cos\left(\frac{19}{18}\beta \right)$
b[15]	$\frac{1}{2}\sqrt{3}\sin\left(\frac{19}{18}\beta \right)$	$-\frac{1}{2}\sin\left(\frac{19}{18}\beta \right)$	$\cos\left(\frac{19}{18}\beta \right)$
b[16]	$\frac{1}{2}\sqrt{3}\sin\left(\frac{17}{18}\beta \right)$	$-\frac{1}{2}\sin\left(\frac{17}{18}\beta \right)$	$\cos\left(\frac{17}{18}\beta \right)$
b[17]	$\frac{1}{2}\sqrt{3}\sin\left(\frac{35}{18}\beta \right)$	$\frac{1}{2}\sin\left(\frac{35}{18}\beta \right)$	$\cos\left(\frac{35}{18}\beta \right)$
b[18]	$-\frac{1}{2}\sqrt{3}\sin\left(\frac{35}{18}\beta \right)$	$\frac{1}{2}\sin\left(\frac{35}{18}\beta \right)$	$\cos\left(\frac{35}{18}\beta \right)$
b[19]	$\frac{1}{2}\sqrt{3}\sin\left(\frac{35}{18}\beta \right)$	$-\frac{1}{2}\sin\left(\frac{35}{18}\beta \right)$	$\cos\left(\frac{35}{18}\beta \right)$
b[20]	$-\frac{1}{2}\sqrt{3}\sin\left(\frac{35}{18}\beta \right)$	$-\frac{1}{2}\sin\left(\frac{35}{18}\beta \right)$	$\cos\left(\frac{35}{18}\beta \right)$

**Table A2:** Nonzero components of the second harmonic generation (SHG) surface tensor with their expressions. α represents the effective SHG surface hyperpolarizability.

Coefficient	Expression
$d_{1113}$	$-\alpha \cos\left(\frac{\pi }{36}\right)\left(2+\cos(2\phi )\right)\sin^{2}\left(\frac{\pi }{36}\right)$
$d_{1123}$	$-\alpha \cos\left(\frac{\pi }{36}\right)\sin^{2}\left(\frac{\pi }{36}\right)\sin\left(2\phi \right)$
$d_{1131}$	$-\alpha \cos\left(\frac{\pi }{36}\right)\left(2+\cos(2\phi )\right)\sin^{2}\left(\frac{\pi }{36}\right)$
$d_{1132}$	$-\alpha \cos\left(\frac{\pi }{36}\right)\sin^{2}\left(\frac{\pi }{36}\right)\sin\left(2\phi \right)$
$d_{1213}$	$-\alpha \cos\left(\frac{\pi }{36}\right)\sin^{2}\left(\frac{\pi }{36}\right)\sin\left(2\phi \right)$
$d_{1223}$	$\alpha \cos\left(\frac{\pi }{36}\right)\left(-2+\cos(2\phi )\right)\sin^{2}\left(\frac{\pi }{36}\right)$
$d_{1231}$	$-\alpha \cos\left(\frac{\pi }{36}\right)\sin^{2}\left(\frac{\pi }{36}\right)\sin\left(2\phi \right)$
$d_{1313}$	$-\alpha \cos\left(\frac{\pi }{36}\right)\left(2+\cos(2\phi )\right)\sin^{2}\left(\frac{\pi }{36}\right)$
$d_{1323}$	$-\alpha \cos\left(\frac{\pi }{36}\right)\sin^{2}\left(\frac{\pi }{36}\right)\sin\left(2\phi \right)$
$d_{1331}$	$-\alpha \cos\left(\frac{\pi }{36}\right)\left(2+\cos(2\phi )\right)\sin^{2}\left(\frac{\pi }{36}\right)$
$d_{1332}$	$-\alpha \cos\left(\frac{\pi }{36}\right)\sin^{2}\left(\frac{\pi }{36}\right)\sin\left(2\phi \right)$
$d_{2113}$	$-\alpha \cos\left(\frac{\pi }{36}\right)\sin^{2}\left(\frac{\pi }{36}\right)\sin\left(2\phi \right)$
$d_{2123}$	$\alpha \cos\left(\frac{\pi }{36}\right)\left(-2+\cos(2\phi )\right)\sin^{2}\left(\frac{\pi }{36}\right)$
$d_{2131}$	$-\alpha \cos\left(\frac{\pi }{36}\right)\sin^{2}\left(\frac{\pi }{36}\right)\sin\left(2\phi \right)$
$d_{2132}$	$\alpha \cos\left(\frac{\pi }{36}\right)\left(-2+\cos(2\phi )\right)\sin^{2}\left(\frac{\pi }{36}\right)$
$d_{2213}$	$\alpha \cos\left(\frac{\pi }{36}\right)\left(-2+\cos(2\phi )\right)\sin^{2}\left(\frac{\pi }{36}\right)$
$d_{2223}$	$-\alpha \cos\left(\frac{\pi }{36}\right)\left(2+\cos(2\phi )\right)\sin^{2}\left(\frac{\pi }{36}\right)$
$d_{2231}$	$\alpha \cos\left(\frac{\pi }{36}\right)\left(-2+\cos(2\phi )\right)\sin^{2}\left(\frac{\pi }{36}\right)$
$d_{2232}$	$-\alpha \cos\left(\frac{\pi }{36}\right)\left(2+\cos(2\phi )\right)\sin^{2}\left(\frac{\pi }{36}\right)$
$d_{2313}$	$\alpha \cos\left(\frac{\pi }{36}\right)\left(-2+\cos(2\phi )\right)\sin^{2}\left(\frac{\pi }{36}\right)$
$d_{2323}$	$\alpha \cos\left(\frac{\pi }{36}\right)\sin^{2}\left(\frac{\pi }{36}\right)\sin\left(2\phi \right)$
$d_{2331}$	$\alpha \cos\left(\frac{\pi }{36}\right)\left(-2+\cos(2\phi )\right)\sin^{2}\left(\frac{\pi }{36}\right)$
$d_{2332}$	$\alpha \cos\left(\frac{\pi }{36}\right)\sin^{2}\left(\frac{\pi }{36}\right)\sin\left(2\phi \right)$
$d_{3113}$	$-\alpha \cos\left(\frac{\pi }{36}\right)\left(2+\cos(2\phi )\right)\sin^{2}\left(\frac{\pi }{36}\right)$
$d_{3123}$	$-\alpha \cos\left(\frac{\pi }{36}\right)\sin^{2}\left(\frac{\pi }{36}\right)\sin\left(2\phi \right)$
$d_{3131}$	$-\alpha \cos\left(\frac{\pi }{36}\right)\left(2+\cos(2\phi )\right)\sin^{2}\left(\frac{\pi }{36}\right)$
$d_{3132}$	$-\alpha \cos\left(\frac{\pi }{36}\right)\sin^{2}\left(\frac{\pi }{36}\right)\sin\left(2\phi \right)$
$d_{3213}$	$-\alpha \cos\left(\frac{\pi }{36}\right)\sin^{2}\left(\frac{\pi }{36}\right)\sin\left(2\phi \right)$
$d_{3223}$	$\alpha \cos\left(\frac{\pi }{36}\right)\left(-2+\cos(2\phi )\right)\sin^{2}\left(\frac{\pi }{36}\right)$
$d_{3231}$	$-\alpha \cos\left(\frac{\pi }{36}\right)\sin^{2}\left(\frac{\pi }{36}\right)\sin\left(2\phi \right)$
$d_{3232}$	$\alpha \cos\left(\frac{\pi }{36}\right)\left(-2+\cos(2\phi )\right)\sin^{2}\left(\frac{\pi }{36}\right)$
$d_{3313}$	$-\alpha \cos\left(\frac{\pi }{36}\right)\left(2+\cos(2\phi )\right)\sin^{2}\left(\frac{\pi }{36}\right)$
$d_{3323}$	$-\alpha \cos\left(\frac{\pi }{36}\right)\sin^{2}\left(\frac{\pi }{36}\right)\sin\left(2\phi \right)$
$d_{3331}$	$2\alpha \cos\left(\frac{\pi }{36}\right)\sin^{4}\left(\frac{\pi }{36}\right)$
$d_{3332}$	$-\alpha \cos\left(\frac{\pi }{36}\right)\sin^{2}\left(\frac{\pi }{36}\right)\sin\left(2\phi \right)$
$d_{3333}$	= $2\alpha \cos\left(\frac{\pi }{36}\right)\sin^{4}\left(\frac{\pi }{36}\right)$
